# PERK-Mediated Suppression of microRNAs by Sildenafil Improves Mitochondrial Dysfunction in Heart Failure

**DOI:** 10.1016/j.isci.2020.101410

**Published:** 2020-07-24

**Authors:** Takashi Shimizu, Akashi Taguchi, Yoshiki Higashijima, Naoko Takubo, Yasuharu Kanki, Yoshihiro Urade, Youichiro Wada

**Affiliations:** 1 Isotope Science Center, The University of Tokyo, Tokyo 113-0032, Japan; 2 Department of Cardiovascular Medicine, Graduate School of Medicine, The University of Tokyo, Tokyo 113-8655, Japan; 3 Department of Bioinformational Pharmacology, Tokyo Medical and Dental University, Tokyo 113-8510, Japan; 4 Department of Proteomics, The Novo Nordisk Foundation Center for Protein Research, Faculty of Health and Medical Sciences, University of Copenhagen, Blegdamsvej 3B, Copenhagen 2200, Denmark

**Keywords:** Biological Sciences, Molecular Biology, Cell Biology

## Abstract

Oxidative/nitrosative stress is a major trigger of cardiac dysfunction, involving the unfolded protein response and mitochondrial dysfunction. Activation of nitric oxide-cyclic guanosine monophosphate-protein kinase G signaling by sildenafil improves cardiac mal-remodeling during pressure-overload-induced heart failure. Transcriptome analysis was conducted in failing hearts with or without sildenafil treatment. Protein kinase R–like endoplasmic reticulum (ER) kinase (PERK) downstream signaling pathways, EIF2 and NRF2, were significantly altered. Although EIF2 signaling was suppressed, NRF2 signaling was upregulated, inhibiting the maturation of miR 24-3p through EGFR-mediated Ago2 phosphorylation. To study the effect of sildenafil on these pathways, we generated cardiac-specific PERK knockout mice. In these mice, sildenafil could not inhibit the maturations, the nuclear translocation of NRF2 was suppressed, and mitochondrial dysfunction advanced. Altogether, these results show that PERK-mediated suppression of miRNAs by sildenafil is vital for maintaining mitochondrial homeostasis through NRF2-mediated oxidative stress response.

## Highlights

• RBM3 inhibited the PERK arm of UPR in chronic heart failure (HF)
• Sildenafil significantly altered PERK downstream signaling pathways, EIF2 and NRF2
• PERK-mediated miRNA suppression by sildenafil was vital for mitochondrial homeostasis
• Ago2 phosphorylation by EGFR was vital for inhibiting HF-induced miRNA maturation

## Graphical Abstract

**Figure:**
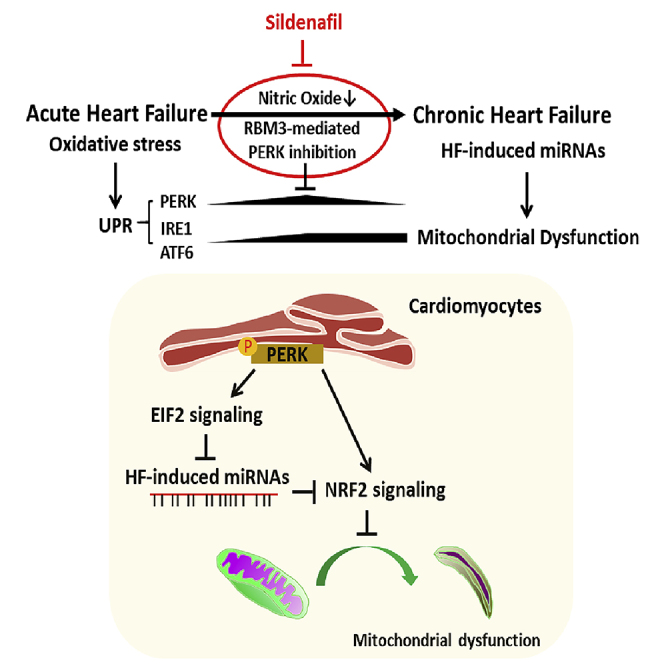

## Introduction

Death due to heart failure (HF) is steadily increasing worldwide. Approximately half of all patients with HF have a preserved ejection fraction (HFpEF), whereas the others have a reduced ejection fraction (HFrEF) (Abebe et al., 2016). An increase in stimuli such as oxidative-nitrosative stress, hypoxia, and mechanical stress results in decompensation in congestive HF. Chronic inflammation related to obesity and hypertension induces excessive production of reactive oxygen species (ROS) in mitochondria, leading to mitochondrial DNA (mtDNA) damage, impairment of sarcomere contraction, and activation of various signaling pathways related to cardiac hypertrophy and apoptosis (Tsutsui et al., 2011). ROS overproduction causes the misfolding of proteins in the ER, which leads to ER stress (Fujii et al., 2018). Three ER stress sensors, inositol-requiring protein-1 (*IRE1*) *α*, activating transcription factor-6 (*ATF6*), and protein kinase RNA (PKR)-like ER kinase (*PERK*) then initiate the unfolded protein response (UPR). Each of the three arms of the UPR, *IRE1α* (Steiger et al., 2018), *ATF6* (Blackwood et al., 2019), and *PERK* (Liu et al., 2014), is known to be protective for hearts exposed to pressure overload (PO).

In cardiomyocytes, cGMP is produced through nitric oxide (NO) stimulation of guanylyl cyclase-1 (GC-1) and natriuretic peptide (NP) stimulation of GC-2A (Lee et al., 2015). An inhibitor of phosphodiesterase type 5 (PDE5-I) is coupled to NO- cyclic guanosine monophosphate (cGMP)-protein kinase G (PKG) signaling, whereas an inhibitor of phosphodiesterase type 9 (PDE9-I) is coupled to NP-cGMP-PKG signaling. Low myocardial PKG activity in HFpEF was associated with low NO bioavailability compared with HFrEF (van Heerebeek et al., 2012). However, the underlying molecular mechanisms remain unknown.

MicroRNAs (miRNAs) are small ribonucleic acids that control mRNA translation and degradation post-transcriptionally, allowing them to be potential biomarkers in differentiating between HFpEF and HFrEF (Watson et al., 2015). Recently, it was reported that PDE5-I, but not PDE9-I, suppressed the maturation of PO-induced miRNAs (Kokkonen-Simon et al., 2018). However, the mechanism by which PDE5-I-coupled NO-cGMP-PKG signaling affects this maturation was not elucidated. Epidermal growth factor receptor (EGFR) suppresses the maturation of some hypoxia-induced miRNAs through the phosphorylation of argonaute 2 (AGO2) at Tyr 393 on stress granules (SGs), which are RNA-containing granules formed in response to the phosphorylation of the α subunit of eukaryotic initiator factor 2 (eIF2α) (Shen et al., 2013) (Pare et al., 2011). Mature miRNAs bind to target mRNAs with sequence complementarity, and this duplex is cleaved by AGO2 with RNA-induced silencing complex. Akt-mediated phosphorylation of AGO2 at Ser387 facilitates its interaction with GW182 and localization to cytoplasmic processing bodies (P bodies), where miRNA-targeted mRNAs are thought to be degraded (Horman et al., 2013). SGs can interact with P bodies, so a similar mechanism of action on SGs may occur on p bodies.

In summary, we hypothesized that sildenafil, one of PDE5-Is, may affect the maturation of PO-induced miRNAs through PERK downstream signaling. PERK has two downstream signaling pathways, EIF2 signaling, related to protein translation and apoptosis, and NRF2 signaling, related to oxidative stress response and mitochondrial homeostasis (Hetz and Papa, 2018). To test this, we broadly analyzed the expression of mRNA and miRNA in HF with or without sildenafil treatment using cardiac-specific PERK knockout (KO) mice.

## Results

### The PERK Arm of UPR Was Suppressed in Hearts Exposed to Chronic PO

We monitored the UPR status in hearts exposed to 3-week (acute) or 7-week (chronic) trans-aortic constriction (TAC, T) (Figure S1A). Phosphorylation of PERK (p-PERK) was upregulated during the acute phase but downregulated during the chronic phase. The expression of ATF4 was inhibited during the chronic phase. The activity of the IRE1α-XBP1 arm was not suppressed in the chronic phase when compared with that in the acute phase. The activity of the ATF6 arm was slightly decreased in the chronic phase when compared with that in the acute phase. The mRNA expression levels of *ATF4* and *CHOP*, genes downstream of the *PERK* arm, were also decreased in hearts exposed to chronic PO compared with those in hearts exposed to Sham (Figure S1B). On the other hand, the mRNA expression levels of *RBM3*, which disturbs the autophosphorylation of PERK (Zhu et al., 2016), and *ANP*, one of the HF-induced NPs, were increased in the chronic phase.

### Sildenafil Suppresses PERK Signaling Though RBM3

To clarify the relationship between cGMP-PKG signaling and *RBM3* expression, we treated NRCMs with Br cGMP (Figure S1C). *RBM3* expression was significantly increased by this treatment. To study the effect of sildenafil on PERK signaling under ER stress *in vitro*, we transfected neonatal rat cardiomyocytes (NRCMs) with control siRNA (Control-SI) or PERK siRNA (PERK-SI) (Figure S1D). Thapsigargin (TG, 1 μM, 24 h) stimulation induced UPR and promoted the phosphorylation of eIF2α in Control-SI cells, but not in PERK-SI cells (Figure S1E). p-eIF2α was suppressed in Control-SI NRCMs treated with TG + sildenafil (sil 1 μM, 24 h) compared with that in those treated with TG and was completely suppressed in PERK-SI NRCMs treated with TG or TG + S.

Next, to investigate the correlation between RBM3 and PERK during sildenafil treatment, we used HEK293T cells transfected with a LacZ plasmid (LacZ) or a flag-tagged RBM3 coding plasmid (Flag-RBM3) (Figures S1F and S1G). Sildenafil promoted p-PERK in LacZ cells but not in Flag-RBM3 cells. Furthermore, we performed luciferase reporter assays using cells transfected with a luciferase reporter plasmid containing the 5′ UTR of ATF4. The luciferase activity was markedly increased upon treatment with 0.1 or 1 μM sildenafil in LacZ cells but that was decreased in Flag-RBM3 cells.

Taken together, sildenafil promoted PERK signaling without RBM3 overexpression but suppressed it under RBM3 overexpression.

### Sildenafil Improves PO-Induced HF through the PERK Arm

**Figure 1:**
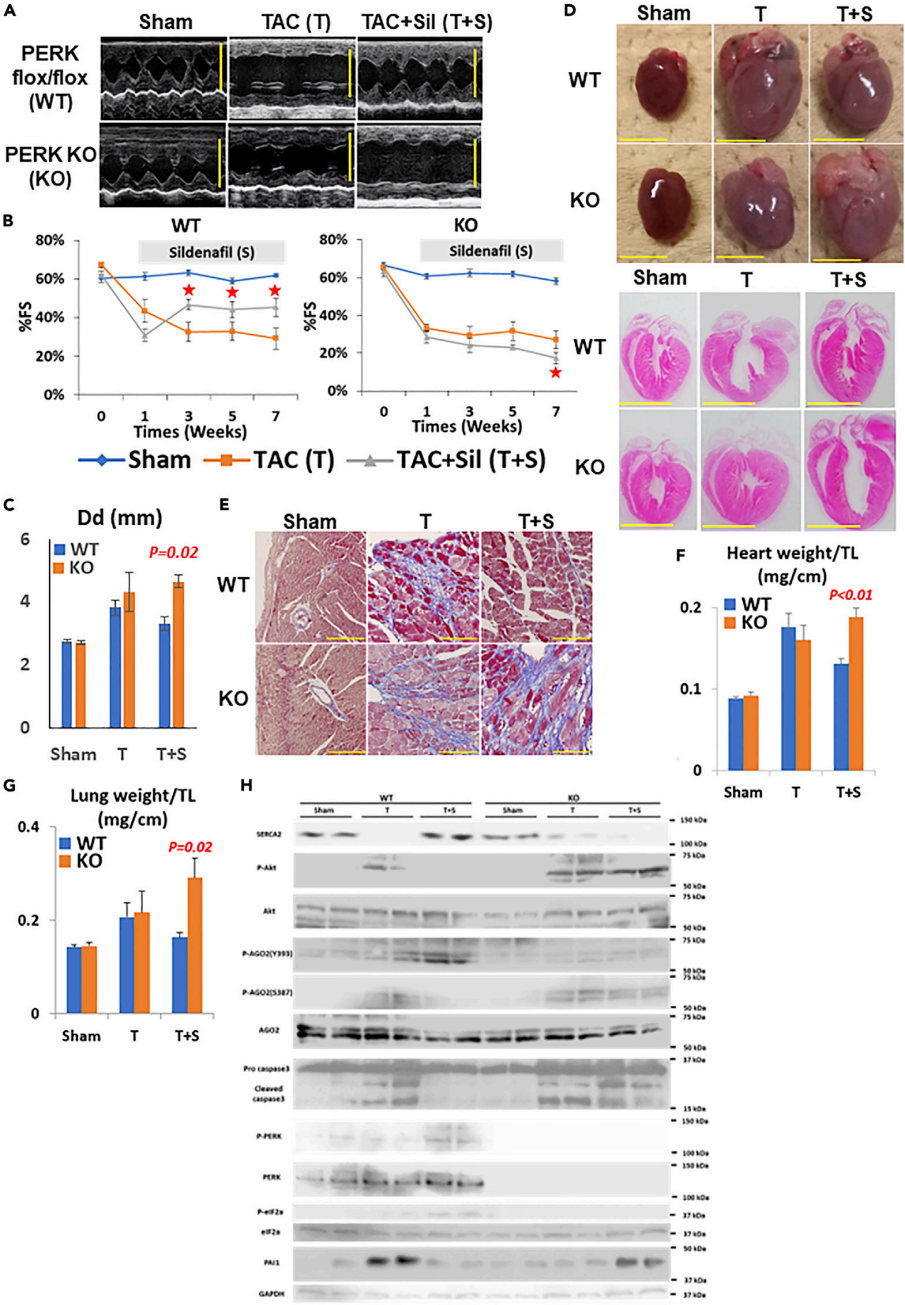
Sildenafil Did Not Improve Pressure Overload (PO)-Induced Heart Failure (HF) in PERK Conditional Knockout (KO) Mice (A) M-mode echocardiograms from PERK flox/flox (wild type, WT) or PERK KO (KO) mice exposed to 7 weeks of Sham, trans-aortic constriction (TAC, T), or sildenafil starting one week after TAC (T + S). Scale bar, 5 mm. (B) Sildenafil reversed left ventricular (LV) percentage of fractional shortening (%FS) in WT mice at the time points of 3, 5, and 7 weeks after TAC. In KO mice, Sildenafil exacerbated %FS at the time point of 7 weeks after TAC. Mean ± SEM was analyzed by un-paired t test. n = 6-11 per group. ★p < 0.05 by un-paired t test versus at the time point of 1 week after TAC. (C) LV diastolic diameter (Dd) (n = 6–10 per group). Mean ± SEM was analyzed by un-paired t test. (D) Example of whole heart (upper) and heart sections stained with Hematoxylin-Eosin (H&E, lower) in each group. Scale bar, 5 mm. (E) Heart sections stained with azan in each group. Scale bar, 100 mm. (F and G) Heart (F) and lung (G) weight normalized to tibial length (TL) (n = 6–10 per group). Mean ± SEM was analyzed by un-paired t test. (H) Western blot analysis of hearts (n = 2 per group).

After 7 weeks of TAC (T), PERK flox/flox (wild type, WT) mice and KO mice developed marked chamber dysfunction (Figures 1A and 1B), dilation, hypertrophy, and fibrosis (Figures 1C–1E). Sildenafil treatment recovered the percentage of fractional shortening (%FS) and left ventricular (LV) dilatation in WT mice but not in KO mice (Figures 1A–1C). Sildenafil also reduced interstitial fibrosis and heart and lung weights in WT mice exposed to TAC (T + S) compared with those in KO mice (Figures 1E–1G).

### Sildenafil Phosphorylates AGO2 at Tyr393 through PERK and EGFR

Sildenafil attenuated the phosphorylation of Akt and AGO2 at Ser387 (S387) and the expression of cleaved caspase-3, a marker for apoptosis, in WT mice withTAC-induced PO but not in KO mice (Figure 1H). Sildenafil increased the expression of sarcoplasmic reticulum calcium uptake pump (SERCA) 2a, PERK, and eIF2α as well as the phosphorylation of AGO2 (p-AGO2) at Tyr393 (Y393) in WT mice but not in KO mice.

In Control-SI cells, the phosphorylation of Akt and AGO2 at Ser387 was suppressed by TG + sil treatment, compared with TG treatment. However, these effects were not observed in PERK-SI cells (Figure S1E). p-AGO2 (Y393) and the phosphorylation of EGFR (p-EGFR) at Thr678 were increased by TG + sil or Br cGMP treatment in Control-SI cells compared with those in TG-treated cells. In PERK-SI cells, p-EGFR was also upregulated by TG + sil or Br cGMP, although p-AGO2 (Y393) was not increased as much as in Control-SI cells. Thus, sildenafil or Br cGMP could not induce p-AGO2 (Y393) without PERK.

We next tried to investigate the mechanism by which Br cGMP or sildenafil affected p-AGO2 (Y393), irrespective of p-EGFR (Figures S2A and S2B). We transfected NRCMs with LacZ, GFP-tagged EGFR (WT), or mutated EGFR T678A (non-phosphorylatable mutant) plasmids. Br cGMP or sildenafil stimulation increased p-EGFR and p-AGO2 (Y393) in cells transfected with EGFR. However, Br cGMP or sildenafil stimulation did not affect cells transfected with EGFR T678A. Therefore, Br cGMP or sildenafil could not induce p-AGO2 (Y393) without p-EGFR.

### The Phosphorylation of AGO2 at Tyr393 Occurred on SGs

Recently, it was shown that AGO2 phosphorylation (Y393) by EGFR suppresses the maturation of some miRNAs on SGs, which are RNA-containing granules formed in response to phosphorylation of eIF2α (Pare et al., 2011). To examine the molecular mechanism by which PERK inhibition suppresses p-AGO2 (Y393) by EGFR on SGs, we transfected a flag-tagged AGO2 coding plasmid into NRCMs and added various stimulations to them. Then, we conducted immunoprecipitation (IP) with the anti-FLAG antibody for cell lysates of these cells (Figure S2C). AGO2 phosphorylation (Y393) occurred in both total cell lysates and IP lysates under TG + sil or Br cGMP but not under TG + PERKI or Br cGMP + PERKI. G3BP1 (a marker for SGs) was detected in IP lysates under TG, TG + sil, or Br cGMP. EGFR phosphorylation occurred in total cell lysates under TG + sil, Br cGMP, or Br cGMP + PERKI but not in IP lysates under Br cGMP + PERKI. Furthermore, EGFR itself was not detected in IP lysates under TG + PERKI or Br cGMP + PERKI. As a result, we hypothesized that PERK might be necessary for the formation of SGs and the binding of AGO2 to EGFR on SGs. To investigate this, we performed colocalization assays of G3BP1 and EGFR in Control-SI or PERK-SI NRCMs (Figure S2D). In Control-SI cells, the formation of SGs and EGFR–SG colocalization occurred under TG, TG + sil, and Br cGMP stimulations. On the other hand, SGs were not formed in PERK-SI cells under any stimulations, and EGFR was also not co-localized. Overall, the formation of SGs by PERK signaling may be vital for the phosphorylation of AGO2 at Tyr393 by EGFR on SGs.

### Sildenafil Upregulates NRF2-Mediated Oxidative Stress through the PERK Arm

**Figure 2:**
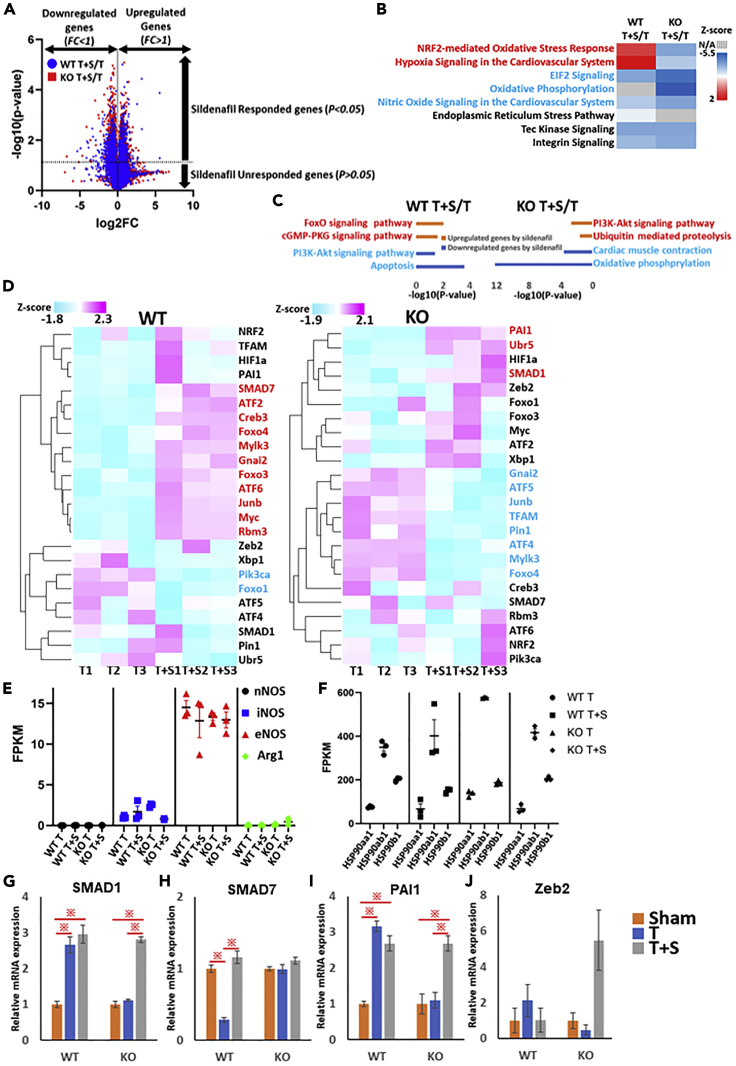
Comparison of Hearts Exposed to T or T + S with or without PERK Inhibition, Using RNA-Sequencing LV myocardium from WT and KO mice subjected to TAC surgery and subsequently given either vehicle or sildenafil were subjected to RNA-sequencing and subsequent differential expression analysis (n = 3 per group). (A) Volcano plot of RNA sequencing data, depicting mRNA data p values, calculated using the un-paired t test, versus fold change (FC). Genes, whose expressions were significantly (p < 0.05) changed by sildenafil, were defined as the sildenafil responded. Next, we defined upregulated genes by sildenafil as FC > 1 and p < 0.05 and downregulated genes by sildenafil as FC < 1 and p < 0.05. Blue circles mean genes in T + S/T of WT mice, and red squares mean genes in T + S/T of KO mice. (B) Summary of the canonical pathways predicted by Ingenuity Pathway Analysis (IPA) in the comparison of sildenafil responded genes in T + S/T of WT or KO mice. Canonical pathway analysis identified pathways, from the IPA library of canonical pathways that were most significant to the dataset. The representative pathways, related to PERK signaling HF and NO-cGMP-PKG signaling, were depicted using activation z-scores. Red means upregulated pathways by sildenafil in WT mice. Blue means downregulated pathways by sildenafil in KO mice. Black means unchanged pathways between WT and KO mice. (C) Summary of the Kyoto Encyclopedia of Genes and Genomes (KEGG) pathway analysis for upregulated or downregulated genes by sildenafil in WT or KO mice, using the Functional Annotation tool at DAVID Bioinformatics Resources 6.7. The representative pathways were expressed, using −log10(p value), which was calculated via a Fisher's exact value. (D) Expression profiles for representative genes at (A) were summarized in the heatmap, using activation z-scores. Red means upregulated genes by sildenafil. Blue means downregulated genes by sildenafil. Black means unchanged genes by sildenafil. (E and F) The FPKM values for NO synthetase (NOS)-related genes (E) and HSP90-consisted genes (F) in RNA-sequencing (n = 3 per group). (G–J) The expressions of SMAD1 (G), SMAD7 (H), PAI1 (I), and Zeb2 (J); all normalized to GAPDH (n = 3–5 per group). ※p < 0.05, one-way ANOVA with Bonferroni correction.

RNA-sequencing was performed to understand how the transcriptome changes with sildenafil treatment in WT or KO mice exposed to TAC. Each analysis was performed in whole myocardial tissue isolates obtained at terminal study (7 weeks after TAC). All RNA-seq results are provided in Table S1. A total of 9,477 genes were expressed above five reads in one of the replicates in all groups. Genes whose expressions were significantly (P < 0.05) changed by sildenafil were defined as sildenafil responded (Figure 2A). For the canonical pathway analysis, the Ingenuity Pathway Analysis (IPA) software was used. Representative canonical pathways for sildenafil-responded genes in WT or KO mice are depicted in Figure 2B. Sildenafil upregulated NRF2-mediated oxidative stress response and hypoxia signaling in the cardiovascular system of WT mice but downregulated them in that of KO mice. Sildenafil strongly inhibited EIF2 signaling, oxidative phosphorylation, and NO signaling in the cardiovascular system of KO mice compared with those in that of WT mice. The expression of hypoxia-inducible factor 1-alpha (*HIF1a*), a master transcriptional regulator of cellular response to hypoxia, was not affected by sildenafil in WT and KO mice (Figure 2D). However, although the activators of *HIF1a*, such as *JunB* (Alfranca et al., 2002) and *Creb3* (Segarra-Mondejar et al., 2018), were increased by sildenafil in WT mice, they were decreased or not changed in KO mice.

Sildenafil did not affect the whole ER stress pathway itself (Figure 2B). We evaluated key genes related to each arm of the UPR (Figure 2D). The expressions of *ATF4* and *ATF5*, related to the PERK arm, were suppressed in KO mice but not in WT mice. The expression of XBP1, related to the IRE1 arm, was not changed by sildenafil in WT and KO mice. The expression of ATF6, which controls the ATF6 arm, was upregulated by sildenafil in WT mice but not in KO mice. The expression of XBP1 was not changed by sildenafil in WT and KO mice. The expression of ATF6, which controls one arm of the UPR, was upregulated by sildenafil in WT mice but not in KO mice. Therefore, sildenafil induces the PERK and ATF6 arms but not the IRE1 arm.

Both EIF2 signaling and NRF2 signaling are related to the PERK arm. However, in this analysis, the changes in both signaling pathways by sildenafil were different in WT mice but not in KO mice.

### Sildenafil Maintains Mitochondrial Homeostasis through PERK

**Figure 3:**
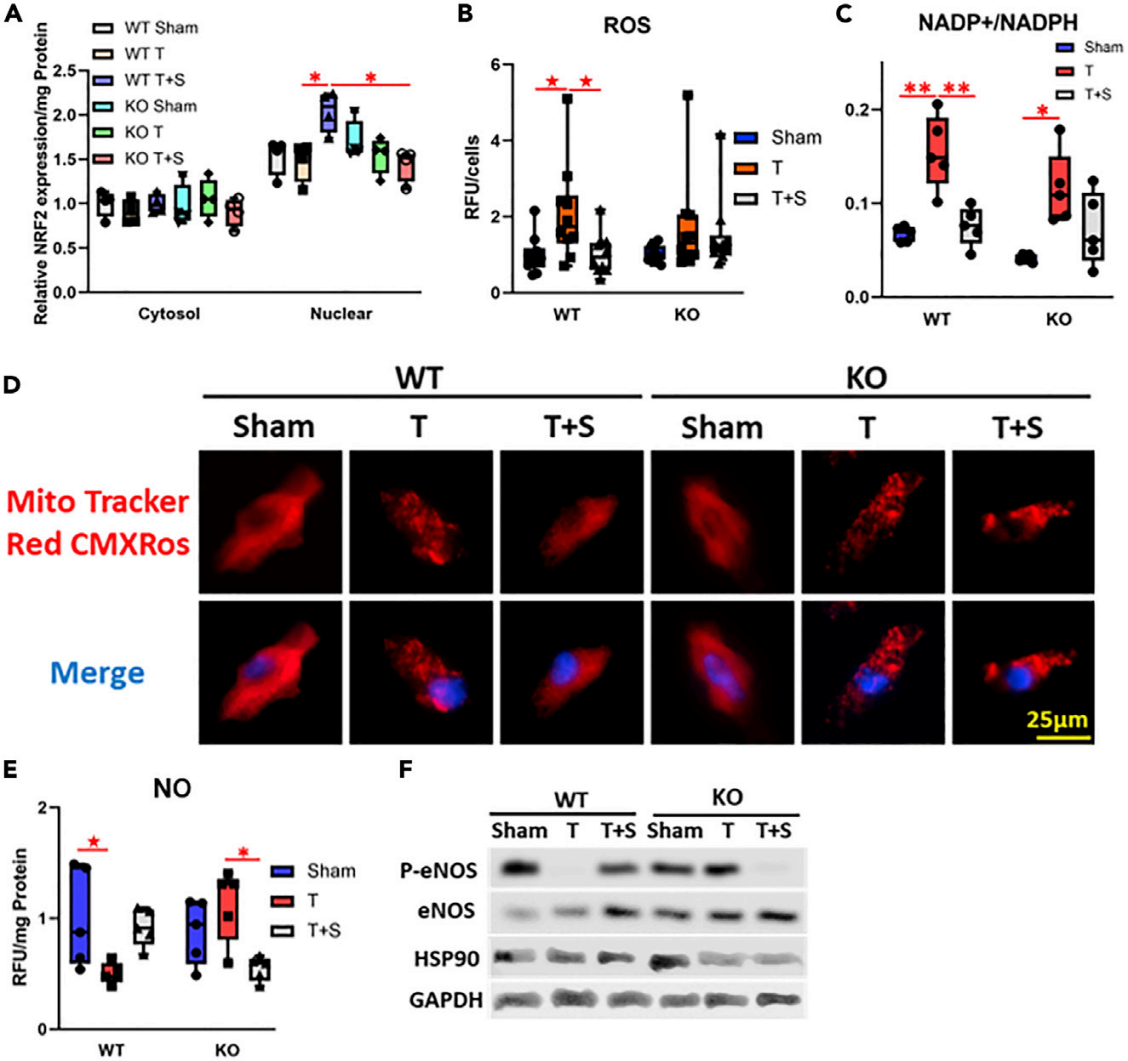
Sildenafil Improves Mitochondrial Function by Upregulating eNOS Activity and Promoting NRF2 Nuclear Translocation via PERK In Figure 2B, NRF2-mediated oxidative stress response was activated by sildenafil in WT mice, but oxidative phosphorylation and nitric oxide signaling were repressed by sildenafil in KO mice. To check how PERK affects the expressions of NO and ROS, mitochondrial morphology, and function, we analyzed hearts or isolated adult cardiomyocytes. (A) The levels of NRF2 in cytosolic and nuclear fractions of isolated cardiomyocytes were analyzed by ELISA (n = 4 per group). ∗p < 0.01 one-way ANOVA with Bonferroni correction. (B) The levels of ROS in isolated cardiomyocytes were assessed by ELISA (n = 10 wells per group). ★p < 0.05, two-way ANOVA with Bonferroni correction. (C) The ratio of NADP + to NADPH in hearts were assessed by ELISA (n = 5 per group). ★p < 0.05, ∗p < 0.01 two-way ANOVA with Bonferroni correction. (D) Colocalization images of Hoechst (blue) with MitoTracker Red CMXRos (Red) in isolated cardiomyocytes. Mitochondria were stained with MitoTracker Red CMXRos, and the nucleus was stained with Hoechst. Scale bar, 25 μm. (E) The levels of NO in hearts were assessed by ELISA (n = 4 per group). ★p < 0.05, ∗p < 0.01 two-way ANOVA with Bonferroni correction. (F) Western blot analysis of hearts for p-eNOS, NOS, HSP90, and GAPDH.

The expression of *NRF2* was not significantly changed by sildenafil in WT and KO mice (Figure 2D), whereas NRF2-mediated oxidative stress response was markedly promoted in WT mice and suppressed in KO mice (Figure 2B). To examine the effect of sildenafil on the nuclear translocation of NRF2, we conducted ELISA for cytosol and nuclear fractions of hearts from these mice (Figure 3A). Sildenafil treatment did not change NRF2 expression in cytosol fractions but increased it in nuclear fractions of WT mice. However, sildenafil did not change NRF2 expression in either cytosol or nuclear fractions of KO mice. We then investigated ROS levels in isolated cardiomyocytes from these mice (Figure 3B). To measure the levels of NADPH oxidation, the ratios of NADP + to NADPH in mitochondria were measured (Figure 3C). Sildenafil suppressed ROS and oxidative stress in WT mice exposed to TAC but not in KO mice. Therefore, sildenafil could induce NRF2-mediated oxidative stress response in response to PERK-mediated nuclear translocation of NRF2.

Sildenafil also promoted the expression of genes related to mitochondrial homeostasis, such as ATF2, ATF4, ATF5, Creb3, Foxo3, Myc, Pin1, and TFAM through PERK (Figure 2D).

To assess mitochondrial morphology and fragmentation, we performed microscopy analysis for isolated adult cardiomyocytes using MitoTracker Red CMXRos dye (Figure 3D). Mitochondrial fragmentation occurred in WT T and KO mice, which was improved by sildenafil in WT mice but not in KO mice. From the viewpoint of mitochondrial function, oxidative phosphorylation was strongly decreased by sildenafil in KO mice but not in WT mice (Figure 2B). Taken together, sildenafil maintained mitochondrial homeostasis through PERK.

### PERK Is Involved in the Activation of eNOS during Sildenafil Treatment

NO signaling in the cardiovascular system was strongly inhibited by sildenafil in KO mice (Figure 2B), but it remains unknown how PERK is involved in this signaling during sildenafil treatment. Firstly, we checked the levels of NO in the hearts of these mice (Figure 3E). NO expression in WT T mice was suppressed compared with that in WT Sham mice but was not different from that in WT T + S mice. In KO mice exposed to TAC, sildenafil decreased NO level.

Next, we measured the expressions of NO synthases (nNOS, iNOS, and eNOS) and arginase 1 (Arg1, a competitive NOS inhibitor) in RNA-sequencing data (Figure 2E). Among these, the expression of eNOS was the highest and thus eNOS seemed to play a vital role in this signaling. The expression of eNOS was not different among these groups. It is reported that HSP90 and PERK are involved in the activation of eNOS (Carrizzo et al., 2016). The mRNA expression of HSP90 genes, such as *HSP90aa1*, *HSP90ab1*, and *HSP90b1*, was not different among these groups (Figure 2F). HSP90 protein expression was also not different among them (Figure 3F). However, sildenafil promoted the phosphorylation of eNOS (P-eNOS) in WT mice exposed to TAC but suppressed it in KO mice.

Overall, PERK-mediated eNOS activation during sildenafil treatment is vital for this signaling.

### Sildenafil Is Not Able to Suppress PI3K-Akt Signaling Pathway without PERK

Next, genes with a fold change (FC) > 1 and p < 0.05 were considered to be upregulated by sildenafil, whereas those with FC < 1 and p < 0.05 were considered to be downregulated by sildenafil. For these genes, Kyoto Encyclopedia of Genes and Genomes (KEGG) enrichment analysis was performed using the Database for Annotation, Visualization, and Integrated Discovery (DAVID software; http://david-d.ncifcrf.gov). Sildenafil upregulated the FoxO signaling pathway and the cGMP-PKG signaling pathway but downregulated the PI3K-Akt signaling pathway and apoptosis in WT mice (Figure 2C). In KO mice, sildenafil activated the PI3K-Akt signaling pathway and ubiquitin-mediated proteolysis but suppressed cardiac muscle contraction and oxidative phosphorylation.

The expression of phosphatidylinositol-4,5-bisphosphate 3-kinase catalytic subunit α (*Pi3kca*) was inhibited by sildenafil in WT mice but not in KO mice (Figure 2D). *Pi3kca* is one of the subunits of PI3 kinase and activates the PI3K-Akt signaling pathway. Sildenafil suppressed the phosphorylation of Akt in WT mice but not in KO mice (Figure 1H). Therefore, sildenafil suppressed this signaling pathway, thereby inhibiting *PERK-Pi3kca* signaling.

### Sildenafil Induces the FoxO and cGMP-PKG Signaling Pathway through PERK

Sildenafil upregulated the expression of *Foxo3* and *Foxo4* and downregulated that of Foxo1 in WT mice (Figure 2D). Although *Foxo4* was downregulated, the expressions of *Foxo1* and *Foxo3* were not changed by sildenafil in KO mice.

Sildenafil increased the expression of *JunB*, which is transcriptionally controlled by *Foxo3*, in WT mice but decreased it in KO mice. The expressions of *ATF2*, *Creb3*, *Gnai2*, and *Mylk3*, which are related to the cGMP-PKG signaling pathway, were promoted by sildenafil in WT mice. However, the expressions of *ATF2* and *Creb3* remained unchanged and those of *Gnai2* and *Mylk3* were downregulated by sildenafil in KO mice.

### Ubiquitin-Mediated Proteolysis and TGF-β Signaling Were Advanced by Sildenafil without PERK

The expression of *Ubr5*, one of the E3 ubiquitin-protein ligases for ubiquitin-mediated proteolysis, was upregulated by sildenafil in KO mice but not in WT mice (Figure 2D). The expression of *SMAD7*, which inhibits TGF-β signaling, was increased by sildenafil in WT mice, and that of *SMAD1*, which promotes TGF-β signaling, was also increased in KO mice (Figures 2D, 2G, and 2H). The expression of PAI1, a target gene of TGF-β signaling, was not changed by sildenafil in WT mice but was increased in KO mice (Figures 2D and 2I). The protein expression of PAI1 was decreased by sildenafil in WT mice but was increased in KO mice (Figure 1H). The expression of Zeb2, the other target gene of TGF-β signaling, was not changed by sildenafil either in WT or in KO mice (Figures 2D and 2J).

### Hearts Exposed to T + S in WT and KO Mice Show Disparate miRNA Profiles

**Figure 4:**
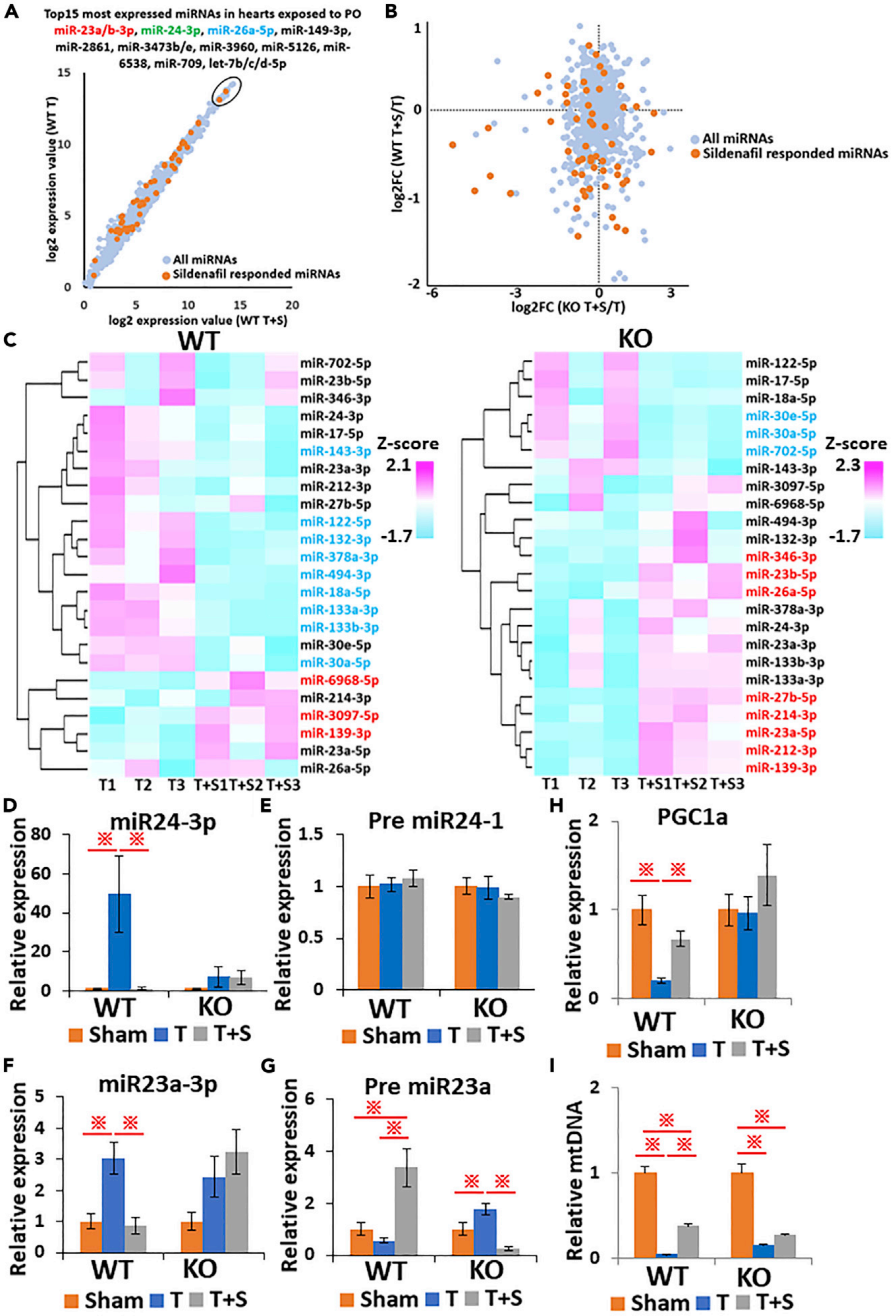
Sildenafil Applied to PO Heart Yield Disparate miRNA Profiles in WT and KO Mice Hearts from WT and KO mice subjected to T or T + S were subjected to miRNA microarray and subsequent differential expression analysis (n = 3 per group). (A) Scatterplot of miRNAs altered in T + S versus T in WT mice, depicting miRNA expression values. The distributions of all miRNAs (gray) and sildenafil-responded miRNAs (orange) were shown. Top 15 most expressed miRNAs in WT T mice were depicted. miR 23a/b-3p (red) and miR 24-3p (green) were PO-induced miRNAs. miR 24-3p and miR 26a-5p (blue) were sildenafil-responded miRNAs. (B) Scatterplot of the distributions of all miRNAs and sildenafil-responded miRNAs between in WT T + S/T and KO T + S/T mice, depicting fold changes of miRNA expression values. (C) Expression profiles for representative miRNAs at (B) were summarized in the heatmap, using activation z-scores. Red means upregulated miRNAs by sildenafil. Blue means downregulated miRNAs by sildenafil. Black means unchanged miRNAs by sildenafil. (D and E) The expressions of miR 24-3p (D) and miR 23a-3p (E); all normalized to U6 snRNA (n = 5 per group). ※p < 0.05, one-way ANOVA with Bonferroni correction. (F–H) The expressions of pre miR 24-1 (F), pre miR 23a (G), and PGC1a (H); all normalized to GAPDH (n = 5 per group). ※p < 0.05, one-way ANOVA with Bonferroni correction. (I) Relative mtDNA quantification of hearts (n = 5 per group). ※p < 0.05, one-way ANOVA with Bonferroni correction.

Figure 4 displays the miRNA microarray results as scatter (Figures 4A and 4B) and heatmap (Figure 4C) plots for two-group comparisons: T + S versus T in WT mice or T + S versus T in KO mice. In WT mice, sildenafil significantly increased 21 miRNAs and decreased 36 miRNAs (Table S2). In KO mice, sildenafil significantly increased 28 miRNAs and decreased 27 miRNAs (Table S2). Many miRNAs that were induced by PO (defined as “HF-induced miRNAs”), such as miR 214-3p, 132-3p, 212-3p, 23a/b-5p, 23a/b-3p, 24-3p, 133a/b-3p, 139-3p, 378a-3p, 27b-5p, and 30e-5p, and some miRNAs suppressed by sildenafil in mice exposed to PO (defined as “sildenafil responded miRNAs”), such as miR 18a-5p, 24-3p, 26a-5p, 30a/e−5p, and 143-3p, were observed in this analysis (Table S3) (Ucar et al., 2012; Lai et al., 2015; Lin et al., 2009; Qu et al., 2016; Kokkonen-Simon et al., 2018).

The scatterplot in Figure 4A depicts the distribution of all miRNAs and sildenafil-responded miRNAs in WT T or T + S mice. The top 15 of the most expressed miRNAs in hearts of WT T mice included some HF-induced miRNAs (miR 23a/b-3p and miR 24-3p) and sildenafil-responded miRNAs (miR 24-3p and miR 26a-5p). The distribution of all miRNAs and sildenafil-responded miRNAs in WT and KO mice is depicted in Figure 4B. Representative miRNAs, whose distributions were changed by PERK deletion, are shown in Figure 4C.

In WT mice, the expression of miR 24-3p, controlled by RBM3 (Pilotte et al., 2011), was upregulated in the hearts exposed to T, when compared with that in Sham or T + S-exposed hearts (Figure 4D). However, in KO mice, the expressions of miR 24-3p and pre miR 24-1 were not changed by sildenafil and these treatments, respectively (Figure 4F).

The expression of miR 23a-3p, inhibiting PGC1a (Wang et al., 2012a), was upregulated in hearts exposed to T compared with that in Sham or T + S-exposed hearts in WT mice but not in KO mice (Figure 4E). The expression levels of pre miR 23a were increased in hearts exposed to T + S compared with those in Sham or T-exposed hearts in WT mice (Figure 4G). In KO mice, the expression levels were increased in hearts exposed to T compared with those in Sham or T + S-exposed hearts. Therefore, some miRNAs could not be maturated by sildenafil without PERK. The mRNA expression of *PGC1a* was downregulated in hearts exposed to T compared with that in Sham or T + S-exposed hearts in WT mice but not in KO mice (Figure 4H). The expression of mitochondrial DNA (mtDNA) was increased in WT mice with T + S compared with those in mice with T (Figure 4I). In KO mice exposed to T, the expression levels of mtDNA were not changed by sildenafil.

### p-AGO2 (Y393) Is Vital for the Suppression of Maturation of Sildenafil-Responded miRNAs

To study how PERK, p-EGFR, and p-AGO2 (Y393) affect the maturation of these miRNAs, we conducted *in vitro* experiments using NRCMs.

**Figure 5:**
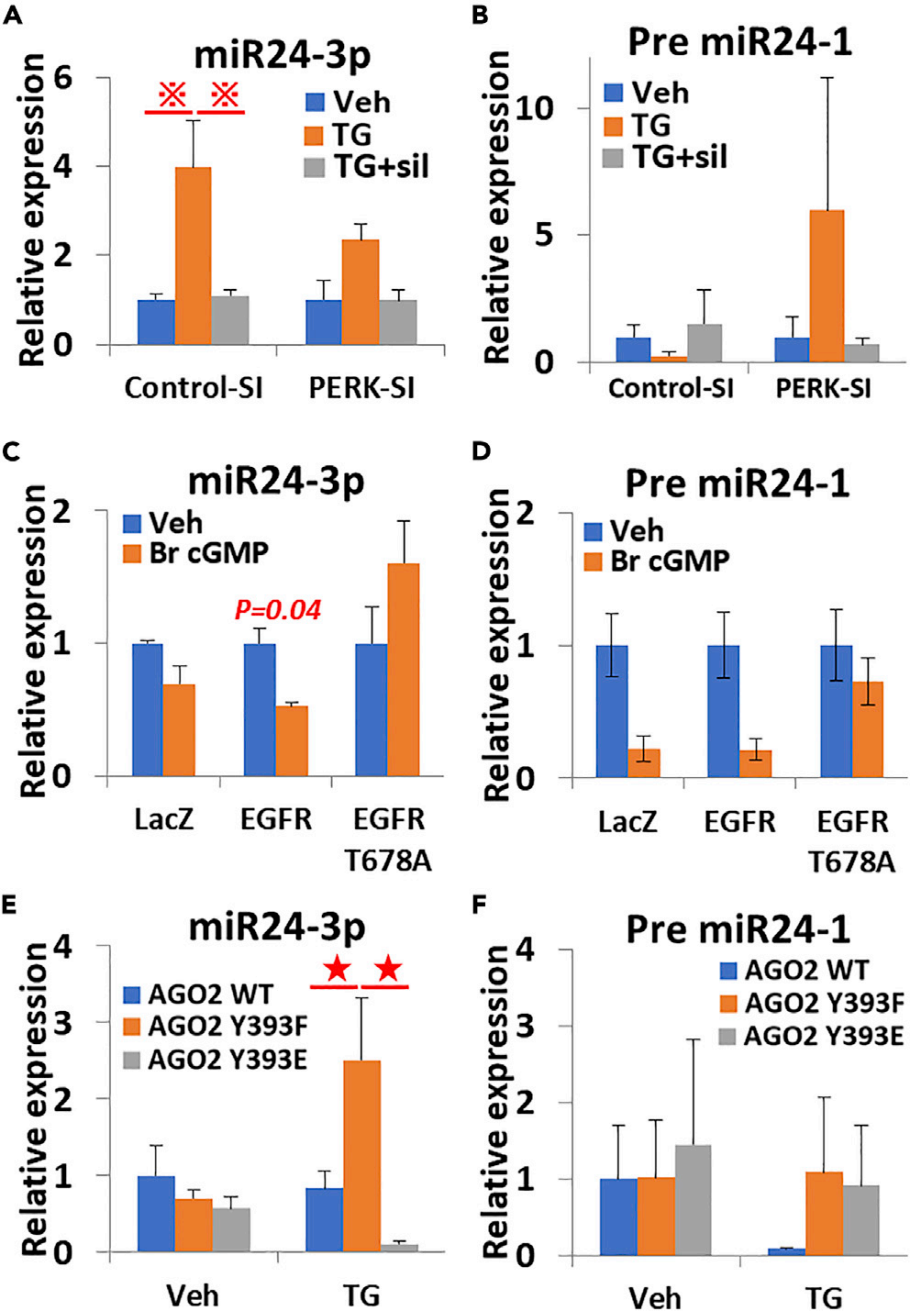
PERK-EGFR-AGO2 Signaling Affected the Maturation of miR 24-3p miR 24-3p is one of PO-induced and sildenafil-suppressed miRNAs. To study how PERK, p-EGFR, and p-AGO2 (Y393) affect the maturation of these miRNAs, we conducted *in vitro* experiments, using neonate rat cardiomyocytes (NRCMs) transfected with siRNAs (control siRNA; Control-SI or PERK siRNA; PERK-SI, A and B) or plasmids (C–F) (n = 3 per group). Plasmids of LacZ, GFP-tagged EGFR, and EGFR T678A were used in (C and D), and those of AGO2 wild type (WT), a mutated AGO2 Y393F (p-AGO2 at Tyr 393 was inhibited), a mutated AGO2 Y393E (p-AGO2 at Tyr 393 was overactivaed) were used in (E&F). (A, C, and E) The expressions of miR 24-3p; all normalized to U6 snRNA (n = 3 per group). (B, D, and F) The expressions of pre miR 24-1; all normalized to GAPDH (n = 3 per group). ※p < 0.05, one-way ANOVA with Bonferroni correction in (A and B). Mean ± SEM was analyzed by un-paired t test in (C and D). ★p < 0.05, two-way ANOVA with Bonferroni correction in (E and F).

The expression of miR24-3p was increased by TG stimulation in Control-SI cells compared with that in vehicle (Veh) or TG + sil-treated cells but not in PERK-SI cells (Figure 5A). The expression of pre miR24-1 was not changed among these cells treated with Veh, TG, or TG + sil (Figure 5B).

The expression of miR24-3p was suppressed by Br cGMP treatment in cells transfected with an EGFR plasmid but not in cells transfected with plasmids of LacZ or EGFR T678A (a non-phosphorylatable mutant) (Figure 5C). The expression of pre miR24-1 was not changed by Br cGMP among these cells (Figure 5D).

Further, the expression of miR24-3p was increased by TG stimulation in cells with mutated AGO2 Y393F compared with that in cells with WT AGO2 or mutated AGO2 Y393E plasmids (Figure 5E). The expression of pre miR24-1 was not changed by TG among these cells (Figure 5F). Taken together, p-AGO2 (Y393) is necessary to suppress the maturation of miR24-3p.

## Discussion

In this study, we have identified PERK to be vital for sildenafil-mediated improvement of mitochondrial dysfunction in failing hearts through the suppression of HF-induced miRNAs. However, PERK has two downstream signaling pathways, EIF2 signaling and NRF2 signaling (Hetz and Papa, 2018). Sildenafil suppressed EIF2 signaling in both WT and KO mice exposed to PO (Figure 2B) due to the genetic deletion of PERK- and RBM3-mediated PERK inhibition by sildenafil in WT mice. The expression levels of *ATF5*, which is a target gene of EIF2 signaling, were suppressed by sildenafil in KO mice but not in WT mice (Figure 2D). This is because ATF4 and ATF5 control an integrated mitochondrial stress response (ISRmt), which consists of one carbon folate cycle and mitochondrial UPR (Khan et al., 2017). The expression of miR 214-3p, which suppressed *ATF4* (Wang et al., 2013), was upregulated by sildenafil in KO mice but not in WT mice (Figure 3B). According to the TargetScan prediction for miRNA-mRNA interactions, miR 346-3p and *ATF5* may have interacted, but further experiments are required to confirm this.

Sildenafil promoted the nuclear translocation of NRF2 through PERK (Figure 3A), resulting in the suppression of ROS production (Figure 3B). Moreover, it was reported that *Pin1* maintained redox balance via synergistic activation of *Myc* and *NRF2* to upregulate the expression of antioxidant response element (ARE)-driven genes (Liang et al., 2019), such as *TFAM*, a key activator of mitochondrial transcription, genome replication, and oxidative phosphorylation (Kang et al., 2018). Sildenafil increased the expression of *Myc*, one of the target genes of EIF2 signaling, in WT mice, and decreased the expression of *Pin1* in KO mice (Figure 2D). Furthermore, sildenafil upregulated NRF2 signaling by inhibiting the maturation of PO-induced miRNAs, such as miR 23a-3p (Figure 4G), miR 24-3p (Figure 4E), and miR 132-3p (Figure 4C). miR24-3p is one of the PO-induced and sildenafil-suppressed miRNAs (Kokkonen-Simon et al., 2018). It is also known that miR 24 suppression prevents the transition from compensated hypertrophy to decompensated hypertrophy in hearts exposed to PO (Li et al., 2013). *Myc*, one of the target genes of miR 24-3p (Lal et al., 2009), directly regulates mitochondrial metabolism and redox homeostasis in hearts exposed to PO, suppressing the expression of miR 23a/b (Ahuja et al., 2010; Gao et al., 2009). miR 132-3p is also known to suppress NRF2 signaling (Zeng et al., 2018). Therefore, reducing oxidative stress and preventing mitochondrial dysfunction through suppression of these miRNAs play a pivotal role in sildenafil-induced cardiac protection.

PO upregulates cardiomyocyte expression of miR-212 and miR-132, which leads to cardiac hypertrophy and dysfunction (Ucar et al., 2012). Both miR-212 and miR-132 directly inhibit anti-hypertrophic and pro-autophagic Foxo3 (Ucar et al., 2012). Sildenafil activated the FoxO signaling pathway in WT mice (Figure 2C). FoxOs, such as Foxo1, 3, and 4, play important roles in cardiac diseases (Xin et al., 2017). *Foxo1* mainly exerts a detrimental effect on the heart by promoting nitrosative stress by upregulating the expression of iNOS. *Foxo3* mainly exerts a beneficial effect on the heart by maintaining mitochondrial homeostasis with PGC1a. *Foxo4* mainly suppresses inflammatory responses and NO production by enhancing the expression of Arg1 (a competitive NOS inhibitor) (Zhu et al., 2015). However, in this study, eNOS was more expressed than did iNOS or Arg1 (Figure 2E). The levels of NO and the phosphorylation of eNOS in the hearts were strongly inhibited by sildenafil in KO mice compared with those in WT mice (Figures 3E and 3F). It is reported that HSP90 and PERK are involved in the activation of eNOS (Carrizzo et al., 2016). In this study, PERK deletion during sildenafil treatment affected the phosphorylation of eNOS but not the expression of HSP90. Therefore, Foxo1&4 may not play a pivotal role in NO production but PERK may do.

Suppression of miR-132-3p by sildenafil in WT mice led to the upregulation of *Foxo3*. However, promotion of miR 212-3p by sildenafil in KO mice led to the downregulation of Foxo3 signaling.

Sildenafil also upregulated the expression of cGMP-PKG target genes, such as *ATF2*, *Creb3*, *Gnai2*, and *Mylk3*, in WT mice (Figures 2C and 2D). *ATF2* upregulates the expression of *PGC1a* (Bordicchia et al., 2012) and increases oxidative phosphorylation in mitochondria. *Creb3* and *JunB*, target genes of Foxo3, activate HIF1a signaling and play anti-apoptotic roles under hypoxia (Flamant et al., 2010). A salient characteristic of dysfunctional myocardium progressing to HF is an upregulation of *Gnai2*, which is involved in GI signaling (DeGeorge et al., 2008). *Gnai2* plays a cardioprotective role, preventing apoptosis. *Mylk3* regulates sarcomere organization and cardiomyocyte contraction and phosphorylates cardiac myosin heavy and light chains (Chan et al., 2008).

Sildenafil inhibited the expression of Pik3ca and the phosphorylation of Akt in WT mice (Figures 1G and 2D) but not in KO mice. It is reported that sildenafil prevents cardiac mal-remodeling, suppressing Akt activity in mice exposed to PO (Takimoto et al., 2005), and P-Akt causes the inactivation of Foxos (Guertin et al., 2006).

The expression of miR 23b (Jiang et al., 2013) or miR 494-3p (Lemecha et al., 2018), which suppressed the expression of TFAM, was not downregulated by sildenafil in KO mice (Figures 4B and 4E). TFAM plays a vital role in mitochondrial genome replication. Therefore, sildenafil could not increase mtDNA expression level in KO mice (Figure 2H).

The expression of *ATF6*, which controls one arm of the UPR, was upregulated by sildenafil in WT mice but not in KO mice. Sildenafil upregulated the expression levels of miR 702-5p, which downregulates *ATF6* (Wang et al., 2016), in KO mice (Figure 3C).

By suppressing TGF-β-SMAD signaling, sildenafil prevents cardiac fibrosis in failing hearts (Gong et al., 2014). The expression of *SMAD7*, which inhibits this signaling and is suppressed by miR 27b-5p (Rong et al., 2018), was increased by sildenafil in WT mice, but that of *SMAD1*, which promotes the signaling and was inhibited by miR 26a-5p (Luzi et al., 2008), was also increased in KO mice. PAI1 is a target gene of TGF-β-SMAD signaling and participates in cardiac-selective fibrosis (Ghosh et al., 2013). Its expression was inhibited by sildenafil in WT mice but advanced in KO mice (Figure 1H). As a result, the suppression of TGF-β signaling or cardiac fibrosis by sildenafil was canceled by PERK deletion.

Some miRNAs (miR 23a/b-5p, 23a/b-3p, 378a-3p, 143-3p, and 27b-5p) are reported to be involved in cardiac hypertrophy, remodeling, and dysfunction (Wang et al., 2012b). Sildenafil inhibited cardiac muscle contraction in KO mice (Figure 2C). Sildenafil also suppressed miR 18a-5p, 378a-3p, and 143-3p in WT mice but enhanced miR 23a/b-5p and 27b-5p in KO mice (Figure 3C). Therefore, sildenafil inhibited these miRNAs through PERK.

The other miRNAs (miR 133a/b-3p and 143-3p) are known to induce apoptosis (Liu et al., 2012; Wang et al., 2014). Sildenafil suppressed these miRNAs in WT mice but not in KO mice (Figure 4C). NO-miR143-induced apoptosis is known to be suppressed by *RBM3* (Yang et al., 2017). Sildenafil upregulated the expression of *RBM3* in WT mice but not in KO mice (Figure 2D). Overall, sildenafil may suppress RBM3-miR143-induced apoptosis through PERK.

miR 17-5p plays a cardioprotective role, and its regulation is able to modulate autophagy. However, the expression of miR 17-5p was not significantly different before and after sildenafil treatment in WT and KO mice (Figure 4C).

In this study, sildenafil induced the phosphorylation of EGFR at Thr678, which is important for keeping internalized EGFR in recycling endosomes and away from degradation pathways (Figure S2D). It is known that EGFR can be phosphorylated at Thr678 by PKC. PKG transmits the cardioprotective signal from the cytosol to the inner mitochondrial membrane, activating PKC (Costa et al., 2005). Thus, PKC plays an intermediate role in the phosphorylation of EGFR at Thr678 during sildenafil treatment.

Stimulation of PKG by sildenafil promotes proteasome-mediated degradation of misfolded proteins (Ranek et al., 2013). In this study, sildenafil enhanced ubiquitin-mediated proteolysis in KO mice (Figure 2C). The expression of genes related to ERAD (ER-associated degradation) is controlled by *XBP1* (Lee et al., 2003). In KO mice, sildenafil did not affect the expression of these genes but upregulated that of the E3 ubiquitin-protein ligase, *Ubr5* (Figure 2D). Therefore, PKG-mediated activation of ubiquitin-mediated proteolysis is not involved in the UPR.

In summary, we have shown that PERK is vital for the cardioprotective effects of sildenafil in PO-induced HF, suppressing the maturation of PO-induced miRNAs, such as miR 23a-3p and 24-3p, and upregulating *NRF2*-mediated oxidative stress response and mitochondrial biogenesis. The interaction between oxidative-nitrosative stress and the UPR is complex (Fujii et al., 2018) but it plays an important role in both HFpEF and HFrEF. In future, according to redox and miRNA profiles, drugs controlling the UPR may be useful for precision therapy.

### Limitations of the Study

PERK-mediated suppression of microRNAs by sildenafil was not checked in human samples. Sildenafil has been shown to improve hemodynamics in patients with HFrEF (Lewis et al., 2007). However, a recent clinical study provided potential adverse effects of sildenafil on mitochondrial function and ER stress in patients with HFpEF (Wang et al., 2017). Therefore, we had better clarify the difference between the effects of sildenafil for HFrEF and HFpEF in the viewpoint of suppression of microRNAs. However, in Japan, it is difficult to get these samples newly, because of COVID-19.

### Resource Availability

#### Lead Contact

Further information and requests for resources and reagents should be directed to and will be fulfilled by the Lead Contact, Takashi Shimizu (tshimizu227-tky@umin.ac.jp)

#### Materials Availability

All data and materials associated with this study are available in the main text or the Supplemental Information.

#### Data and Code Availability

The RNA sequence data can be accessed through the Gene Expression Omnibus (GEO) under the NCBI accession number GSE150481. The microRNA array data can be accessed through the Mendeley Data (https://doi.org/10.17632/d9jzn48bgw.1). The original can be accessed through the Mendeley Data (https://doi.org/10.17632/749tbh7jnt.3).

## Methods

All methods can be found in the accompanying Transparent Methods supplemental file.

## Supplemental Information

Document S1. Transparent Methods and Figures S1 and S2

Table S1. RNA Sequencing Datasets in Hearts of WT or KO Mice Exposed to T or T + S, Related to Figure 2

Table S2. microRNA Microarray Datasets in Hearts of WT or KO Mice Exposed to T or T + S, Related to Figure 4

Table S3. Sildenafil Responded microRNAs in Hearts of WT or KO Mice Exposed to T or T + S, Related to Figure 4

## Footnotes

Supplemental Information can be found online at https://doi.org/10.1016/j.isci.2020.101410.
