# Supplementary material for: PERK-Mediated Suppression of microRNAs by Sildenafil Improves Mitochondrial Dysfunction in Heart Failure
Source: iScience. 2020 Jul 24;23(8):101410. doi: 10.1016/j.isci.2020.101410 (PMC7378464; doi:10.1016/j.isci.2020.101410)
Supplement: Document S1. Transparent Methods and Figures S1 and S2 [file mmc1.pdf]

**iScience, Volume *23***

## Supplemental Information

# PERK-Mediated Suppression of microRNAs by Sildenafil Improves Mitochondrial Dysfunction in Heart Failure

**Takashi Shimizu, Akashi Taguchi, Yoshiki Higashijima, Naoko Takubo, Yasuharu Kanki, Yoshihiro Urade, and Youichiro Wada**

## Supplemental Information

## Transparent Methods

### PERK conditional knockout mice

All experiments were approved by the University of Tokyo Ethics Committee for Animal Experiments and strictly adhered to the guidelines for animal experiments of the University of Tokyo.

To generate mice with cardiomyocyte–specific deletion of PERK (PERK conditional knockout mice, KO mice), mice homozygous for the floxed PERK (WT mice, the Jackson Laboratory, Bar Harbor, ME) were crossed with αMHC-Cre mice, which were gifts from K. Otsu (King's College London). When compared to the WT mice, KO mice have normal cardiac structure and function during unstressed conditions.

### Transverse aortic constriction

As described previously by Takimoto, et al. (2005), PO was performed by surgically placing sutures around the transverse aorta (26G needle size) of the mice. Size-, age-, and sex-matched (male) C57BL/6J mice (CLEA Japan, Inc), WT, or KO mice were subjected to TAC or a Sham operation.

### Isolation of cardiomyocytes from adult mice

The isolation and purification of cardiomyocytes from the adult mice were performed as described before (Ackers-Johnson et al., 2016). Briefly, the cardiomyocytes were isolated from mouse hearts via coronary perfusion with collagenase type 2 (Worthington).

**Biochemical reagents**

Rabbit anti-PERK antibody (1:200), mouse anti-p-eNOS antibody (1:1000), mouse anti-eNOS antibody (1:1000), mouse HSP90 antibody (1:1000), mouse anti-GAPDH antibody (1:1000), mouse anti-XBP1 antibody (1:1000), mouse anti-ATF6 antibody (1:1000), mouse anti-ATF4 antibody (1:1000), control siRNA (Control-SI), PERK siRNA (PERK-SI), and goat anti-G3BP1 antibody were from Santa Cruz Biotechnology (Santa Cruz, CA). Rabbit anti-p-ITPR1 (Ser1756) antibody (1:1000), anti-Akt antibody (1:1000), anti-p-Akt (Ser473) antibody (1:1000), anti-p-PERK (Thr980) antibody (1:100), anti-eIF2α antibody (1:1000), anti-p-eIF2α (Ser51) antibody (1:1000), and anti-cleaved caspase 3 antibody (1:1000) were purchased from Cell Signaling Technology (Beverley, MA). Rabbit anti-p-EGFR (Thr678) antibody (1:100) was purchased from Flarebio Biotech LLC (Baltimore, MD). Rabbit anti-PAI1 antibody (1:1000) was purchased from Cloud-Clone Corp (Katy, TX). Rabbit anti-GFP (Green Fluorescent Protein) antibody was from Medical ＆ Biological Laboratories (Nagoya, Japan). Mouse anti-flag antibody (1:2000) was purchased from Sigma-Aldrich (St. Louis, MO). Rabbit anti-AGO2 antibody (1:1000), anti-p-AGO2 (Tyr393) antibody (1:100), and anti-p-AGO2 (Ser387) antibody (1:100) were purchased from ECM Biosciences (Versailles, KY). 8-bromo-cGMP (Br cGMP) was purchased from Biolog (Bremen, Germany). Thapsigargin (TG) was purchased from Thermo Fisher Scientific (Waltham, MA). Sildenafil was purchased from Wako (Osaka, Japan).

**Total protein extraction from cells and tissue**

Hearts from mice or cultured primary neonatal rat cardiomyocytes (NRCMs) were

homogenized in lysis buffer (Cell signaling technology, #9803) with 1 mM phenylmethylsulfonyl fluoride (PMSF). Isolation and culture of NRCMs were performed as described (Lee et al., 2015). Proteins were denatured in 1X Laemmli buffer by boiling at 100°C for 5 min. The lysates were cooled to room temperature before loading on to western blot gel.

**Western blotting**

Cell or tissue lysate samples, prepared as described above, were resolved on SDS-PAGE. Proteins were then transferred to polyvinylidene fluoride membrane. The membrane was blocked in Tris-buffered saline solution with 0.05% Tween 20 (TBST) and 10% skim milk for 1 h, and then incubated with primary antibody in TBST with 10% skim milk at 4°C overnight. After the incubation, the membrane was washed thrice in TBST and incubated with secondary antibody in TBST with 10% skim milk for 1 h at room temperature. After subsequently washing thrice in TBST, bound antibodies were detected by chemiluminescence with the ECL detection system (GE healthcare).

**Immunoprecipitation**

Cell lysates were incubated with flag antibody overnight at 4°C on gentle rotation. Protein A/G beads (Merck Millipore) were added to the tubes and rotated at 4°C for 1 h. Beads were precipitated by centrifugation at 800 *g* for 30 s and washed thrice with cold lysis buffer. The pellet was resuspended in 2X Laemmli buffer and incubated at 95°C for 5 min. The supernatants were collected and used for western blot analysis.

**RNA isolation, cDNA synthesis, and qPCR**

Total RNA from mouse tissue or cultured NRCMs was isolated with Trizol or miRNeasy micro Kit (Qiagen, Hilden, Germany) with the DNase digestion step, according to the manufacturer's instructions. RNA was used to synthesize cDNA using a cDNA synthesis kit (Thermos Fisher). Quantitative real-time PCR (qRT-PCR) was performed with THUNDERBIRD SYBR qPCR Mix (TOYOBO, #QPS-201), using LightCycler480 (Roche Applied Science). The following SYBR primers were used: rat pre miR 23a (forward 5′-CTGGGGATGGGATTTGCT-3′, reverse 5′-TGGAAATCCCTGGCAATG-3′), rat pre miR 24-1 (forward 5′-CGGTGCCTACTGAGCTGAT-3′, reverse 5′- TCCTGTTCCTGCTGAACTGA-3′), mouse pre miR-23a (forward 5′-CTGGGGATGGGATTTGCT-3′, reverse 5′-GCACAGGGTCAGTTGGAAAT-3′), mouse pre miR 24-1 (forward 5′-CGGTGCCTACTGAGCTGAT-3′, reverse 5′-CGACTCCTGTTCCTGCTGA-3′), mouse *ATF4* (forward 5′-ATGATGGCTTGGCCAGTG-3′, reverse 5′-CCATTTTCTCCAACATCCAATC-3′), mouse *CHOP* (forward 5′-GCGACAGAGCCAGAATAACA-3′, reverse 5′-TCAGGTGTGGTGGTGTATGAA-3′), mouse *PGC-1a* (forward 5′-ACGAAAGGCTCAAGAGGGACGAAT-3′, reverse 5′-CACGGCGCTCTTCAATTGCTTTCT-3′), mouse *SMAD1* (forward 5′-TGAAAACACCAGGCGACATA-3′, reverse 5′- TGAGGCATTCCGCATACAC-3′), mouse *SMAD7* (forward 5′- GAGGCTGCGAGAGGACAC-3′, reverse 5′-GGGCACAGGCTAGTGTGG-3′), mouse *PAI1* (forward 5′-CGTGGCAGCAGGACTGATA-3′, reverse 5′- AGGCCTCTGGGTCATCTACA-3′), mouse *Zeb2* (forward 5′- CATAAATTTGAAGATATTCCCCAATAA-3′, reverse 5′-CATATCCAGGGCTCACAGC-3′), rat *GAPDH* (forward 5′-GACATGCCGCCTGGAGAAAC-3′, reverse 5′-AGCCCAGGATGCCCTTTAGT-3′),

mouse *GAPDH* (forward 5′-CATGGCCTTCCGTGTTCCTA-3′, reverse 5′-CCTGCTTCACCACCTTCTTGAT-3′), mouse *ANP* (forward 5′-CATGGCCTTCCGTGTTCCTA-3′, reverse 5′-CCTGCTTCACCACCTTCTTGAT-3′), mouse *RBM3* (forward 5′-CCGCAGTCTCTCTGTTCTCC-3′, reverse 5′-GTTGAGCCCTCCTACGAAGA-3′), rat *RBM3* (forward 5′-ATATGGGTATGGGCGGTCTA-3′, reverse 5′-TCCTCCTGAGTAGCGGTCAT-3′). All PCR samples were run in duplicate and normalized to GAPDH. Specificity of the SYBR green assays was confirmed by dissociation curve analysis. miRNA was purified using the NucleoSpin® miRNA according to the manufacturer's instructions (Macherey-Nagel, Düren, Germany). Reverse transcription for miRNA was performed using the miScript II RT kit (Qiagen, Toronto, Canada). Quantitative PCR for reverse transcribed miRNA was performed using the miScript SYBR Green PCR kit and specific primer assays for miRNA (Qiagen), using LightCycler480 (Roche Applied Science). The miRNA levels were normalized to U6 (a house keeping gene). Primers for miR 23a-3p, miR 24-3p, and U6 were purchased from Qiagen.

**ELISA**

To assess the levels of NO and NADP/NADPH in hearts, we used NO Colorimetric Assay Kit (Elabscience) and NADP/NADPH Assay Kit-WST (Doujindo). To determine the expression of ROS and NRF2 proteins in isolated adult cardiomyocytes, we used ROS Fluorometric Assay Kit (Elabscience) and NRF2 transcription assay kit (Cayman chemical). To separate cytoplasmic and nuclear protein fractions in isolated adult cardiomyocytes, Cytoplasmic and Nuclear Protein Extraction Kit (BBT) was used.

**miRNA microarray**

The miRNA microarray (Affymetrix GeneChip$^{TM}$ miRNA 4.0 Array) was carried out by Filgen (Aichi, Japan).

**RNA-seq library preparation**

Total RNA from hears from mice was isolated as described above. The RNA integrity score was calculated with the RNA 6000 Nano reagent (Agilent Technologies) in a 2100 Bioanalyzer (Agilent Technologies). RNA-Seq libraries were prepared with a TruSeq RNA Library Prep Kit (Illumina). The libraries were sequenced on a HiSeq 2500 system (Illumina) as single-read 150 base reads.

**mtDNA content**

mtDNA quantitation by qRT-PCR was performed as described (Santulli et al., 2015), comparing with mtDNA 16S rRNA and nuclear DNA (nDNA) β2 microglobulin (B2 MG). The following SYBR primers were used: 16S rRNA (forward 5′-GTTAACCCAACACCGGAATG-3′, reverse 5′-TCTTGTTTGCCGAGTTCCTT-3′), B2 MG (forward 5′-ATGCTGAAGAACGGGAAAAA-3′, reverse 5′-CAGTCTCAGTGGGGGTGAAT-3′).

**Plasmids**

GFP-tagged EGFR and flag-tagged AGO2 plasmids were purchased from Addgene (Cambridge, MA). A Flag-RBM3 plasmid was purchased from Origene (Rockville, MD). Mutation sequences of amino acid substitution (T678A) of GFP-tagged EGFR were designed in-house and commercially constructed by GenScript (Piscataway, NJ).

Flag-tagged AGO2 Y393F and flag-tagged AGO2 Y393E plasmids were generated using the KOD-Plus-Mutagenesis Kit (Toyobo, Japan), according to the manufacturer's instructions. ATF4 luciferase plasmid was constructed in GenScript (Tokyo, Japan) by cloning 413 base pairs of the 5′ UTR of human ATF4 into a pGL4.13[luc2/SV40] vector (Promega).

**Luciferase reporter assay**

Using Lipofectamine 3000, plasmids (150 ng) were transfected into HEK293T cells in a 96-well plate and the luciferase activity was measured using the One-Glo luciferase assay (Promega).

**Chronic drug studies**

For the drug intervention study, which was designed to test the reversal of heart disease established after one week of TAC, mice that were dying in the first week (before drug assignment) or who failed to develop disease after TAC (likely related to inadequate constriction) were excluded from the analysis. WT or KO mice were randomized to receive vehicle or sildenafil (200 mg/kg per day with Bioserv soft diet) initiated one week after TAC. Mice were euthanized at 7 weeks for tissue analysis. Tissue histology and echocardiography followed reported methods (Takimoto et al., 2005).

**Histological analyses and immunostaining**

For histological analyses, mouse hearts were fixed in situ, embedded in paraffin, and stained with hematoxylin & eosin (H&E) or azan staining. Paraffin-embedded heart tissue sections (4-μm thick) were deparaffinized in xylene and rehydrated in a graded

ethanol series.

**Cells and immunohistochemistry**

Transfection of control siRNA (Control-SI, Santa Cruz Biotechnology) or PERK siRNA (PERK-SI, Santa Cruz Biotechnology) into NRCMs was conducted with Lipofectamine 3000 (Thermo Fisher) according to the manufacturer's protocol. Transfection of LacZ, flag-tagged RBM3, GFP-tagged EGFR, EGFR T678A, flag-tagged AGO2, AGO2 Y393F, and AGO2 Y393E plasmids was performed in the same way. For immunohistochemistry, NRCMs were fixed with 50% methanol and 50% acetone, permeabilized with 0.1% saponin in PBS, and blocked in 10% bovine serum albumin in PBS. NRCMs were then incubated overnight with primary antibodies at 4℃ (goat anti-G3BP1 1:100, rabbit anti-EGFR), and then with secondary antibodies for 1 h at room temperature (Alexa Fluor 488- or Alexa Fluor 546-conjugated; Invitrogen), and finally imaged on a fluorescence microscope (FSX100, Olympus Life Science).

To detect mitochondrial morphology and fragmentation,  we used MitoTracker Red CMXRos (Life technologies, M-7512). To this end, isolated adult cardiomyocytes were exposed for 30 min to 200 nM of the MitoTracker Red CMXRos dye in PBS medium supplemented with 10.000× diluted Hoechst dye (Sigma-Aldrich, B2261). Subsequently, cells were washed once with PBS medium, and then imaged on a fluorescence microscope (Leica LAS AF)

**RNA-seq data analysis**

Sequence reads (150-bp single read) were aligned to the mouse reference genome (GRCm38/mm10) with HISAT2 (version: 2.1.0) with default parameters. After

assigning the mapped reads onto the gene positions deposited in the geocode database (https://www.gencodegenes.org/mouse/), the FPKMs (fragments per kilobase of exon per million reads) of all the deposited genes were calculated by CuffLinks with default parameters. RNA-seq signals were visualized with the Integrated Genome Viewer (Version 2.4.8) (http://software.broadinstitute.org/software/igv/). The RNA-seq signal of each locus was normalized on the following basis:

$$Signal\ on\ each\ locus = \frac{Number\ of\ mapped\ \ reads\ on\ each\ locus\ \times\ 1{,}000{,}000}{Total\ number\ of\ mapped\ reads}$$

**Reproducibility between RNA-seq experiments**

The reproducibility of the genome-wide RNA-seq signals in the biological replicates was examined under all conditions. The FPKM values were used as the RNA-seq signals. Subsequently, the correlation coefficients between three biological replicates were calculated based on the FPKMs of each reference gene.

**Volcano plot**

To visualize the effects of sildenafil treatment on mouse hearts exposed to PO, the log2 FCs of mRNA-seq expression levels (horizontal axis) and the -log10 P-values (vertical axis) between TAC+sildenafil (T+S) and TAC (T) in WT or KO mice were plotted. The genes were categorized by the log2 FCs as sildenafil-upregulated genes (FC > 1) and sildenafil-downregulated genes (FC <1).

**Scatter plot**

To visualize the effects of sildenafil treatment on mouse hearts exposed to PO, the log2

FCs of miRNA microarray expression levels between TAC+sildenafil (T+S) and TAC (T) in WT or WT T+S/T and KO T+S/T mice were plotted.

**Signaling pathway analysis**

Functional and canonical pathway analysis was performed for RNA-sequencing data for WT or KO hearts exposed to 7-week TAC (T) or TAC+sildenafil (T+S) using IPA software (Ingenuity® Systems). Comparing T+S to T samples in WT or KO mice, P-values <0.05 in an un-paired t test were selected to define sildenafil responded genes. Among them, genes expressed above 5 reads in one of the replicates in all groups were considered for analysis. FCs of protein expression were computed for the entire expression data set and uploaded for IPA core analysis. Dynamic canonical pathways generated by IPA were curated from specific journal articles, review articles, textbooks, and KEGG Ligand and hand drawn. The significance of the association between the dataset and the canonical pathways was measured using activation z-scores.

**KEGG pathway Analysis**

Among the sildenafil responded genes, we defined upregulated genes as FC>1, and downregulated genes as FC<1. Gene annotation enrichment analysis was performed for KEGG pathway analysis, using the functional annotation tool in DAVID Bioinformatics Resources 6.8 (http://david.abcc.ncifcrf.gov/).

**Statistical analysis**

The data are expressed as mean±SEM. Parametric tests were used after verification to ensure that the variables in each group were normally distributed. Student's un-paired t

tests, as well as one -way or two-way analysis of variance (ANOVA) with Bonferroni correction, were performed using the R software. Nested one-way ANOVA with Bonferroni correction was conducted by Graph Pad Prism 8.4.2. The clustering displayed in the heatmap was also performed using the R software. In RNA-sequencing or miRNA microarray analysis, mRNAs or miRNAs, which were not expressed in hearts exposed to TAC in WT or KO mice, were excluded as we could not calculate the accurate FCs or P-values for these genes. All mRNAs and miRNAs were ranked in a volcano plot according to statistical P-values and relative differences in abundance (FCs). In all tests, differences with P-values $<0.05$ were considered statistically significant.

**Supplemental Reference**

ACKERS-JOHNSON, M., LI, P. Y., HOLMES, A. P., O'BRIEN, S. M., PAVLOVIC, D. & FOO, R. S. 2016. A Simplified, Langendorff-Free Method for Concomitant Isolation of Viable Cardiac Myocytes and Nonmyocytes From the Adult Mouse Heart. Circ Res, 119, 909-20.

LEE, D. I., ZHU, G., SASAKI, T., CHO, G. S., HAMDANI, N., HOLEWINSKI, R., JO, S. H., DANNER, T., ZHANG, M., RAINER, P. P., BEDJA, D., KIRK, J. A., RANEK, M. J., DOSTMANN, W. R., KWON, C., MARGULIES, K. B., VAN EYK, J. E., PAULUS, W. J., TAKIMOTO, E. & KASS, D. A. 2015. Phosphodiesterase 9A controls nitric-oxide-independent cGMP and hypertrophic heart disease. Nature, 519, 472-6.

SANTULLI, G., XIE, W., REIKEN, S. R. & MARKS, A. R. 2015. Mitochondrial calcium overload is a key determinant in heart failure. Proc Natl Acad Sci U S A, 112, 11389-94.

TAKIMOTO, E., CHAMPION, H. C., LI, M., BELARDI, D., REN, S., RODRIGUEZ, E. R., BEDJA, D., GABRIELSON, K. L., WANG, Y. & KASS, D. A. 2005. Chronic inhibition of cyclic GMP phosphodiesterase 5A prevents and reverses cardiac hypertrophy. Nat Med, 11, 214-22.

## Supplemental figures

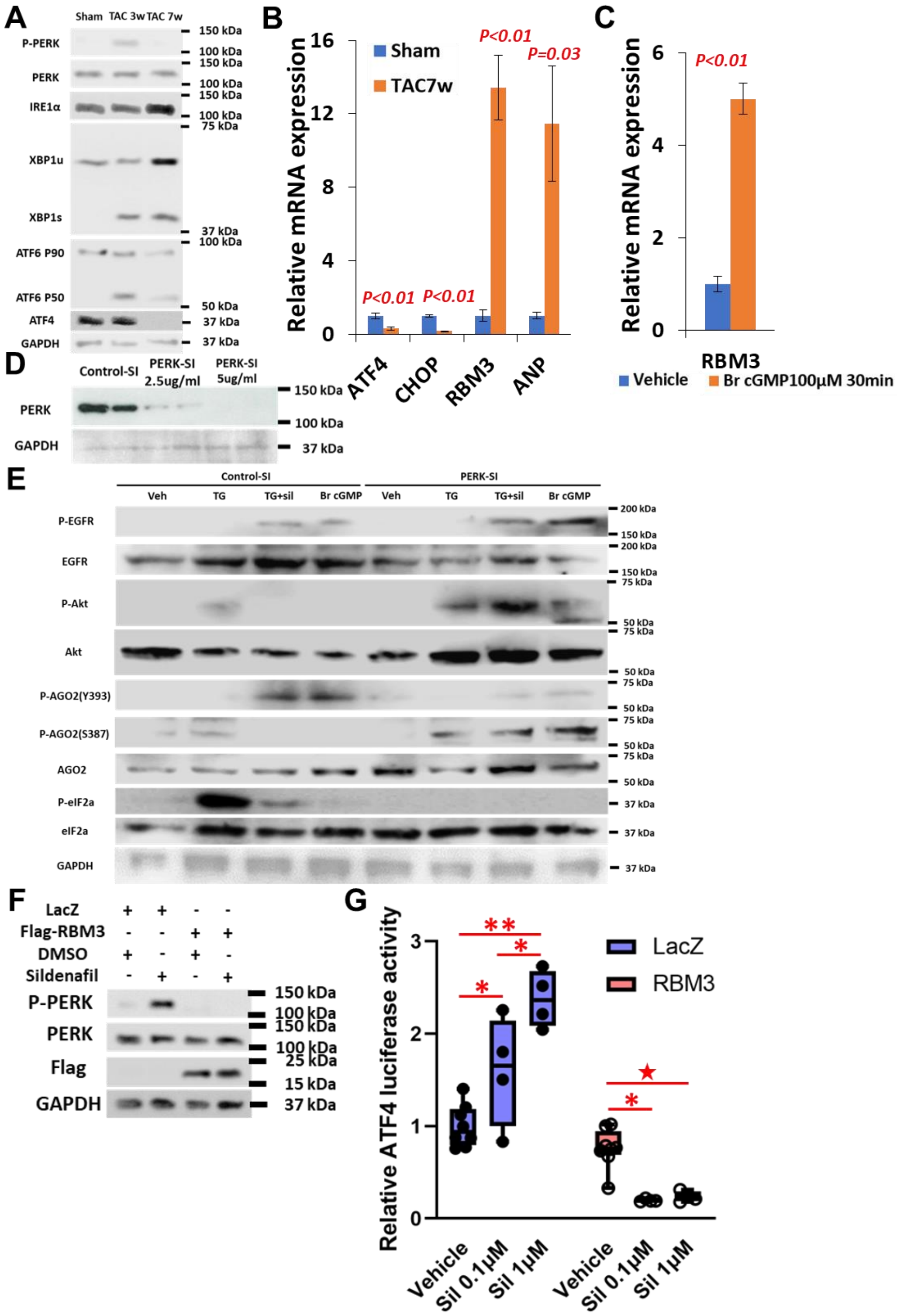


**Figure S1. RBM3 suppressed PERK signaling in chronic phase of HF.**

(A) Western blot analysis of hearts exposed to Sham, 3 or 7week TAC surgery.

(B) The relative mRNA expressions of target genes of EIF2 signaling (ATF4, CHOP), *RBM3*, which inhibits PERK activity, and *ANP*, one of natriuretic peptides induced by HF, in hearts exposed to Sham or 7week TAC ; all normalized to *GAPDH* (n=5 per group). Mean ± SEM was analyzed by un-paired t test.

(C) The relative mRNA expressions of *RBM3* in NRCMs treated with vehicle or 8-bromo-cGMP (Br cGMP, 100μM, 30min) ; all normalized to *GAPDH* (n=3 per group). Mean ± SEM was analyzed by un-paired t test.

(D) Western blot analysis of NRCMs, treated with control siRNA (Control-SI) or PERK siRNA (PERK-SI) 2.5 or 5 μ g/ml.

(E) Western blot analysis of Control-SI or PERK-SI NRCMs, treated with vehicle (Veh), thapsigargin (TG, 1μM 24hr) with or without sildenafil (sil, 1μM 24hr), or Br cGMP (100μM, 30min).

(F) Western blot analysis of HEK293T cells transfected with LacZ (control) or Flag-RBM3 coding plasmid (RBM3), treated with DMSO or sildenafil (1μM 24hr).

(G) Luciferase assay for ATF4 in HEK293T cells with LacZ or RBM3, treated with DMSO, or sildenafil (0.1 or 1μM 24hr). n=4 per group. *P<0.01, **P<0.01, two-way ANOVA with Bonferroni correction.

Figure S1. Related to Figure 2.

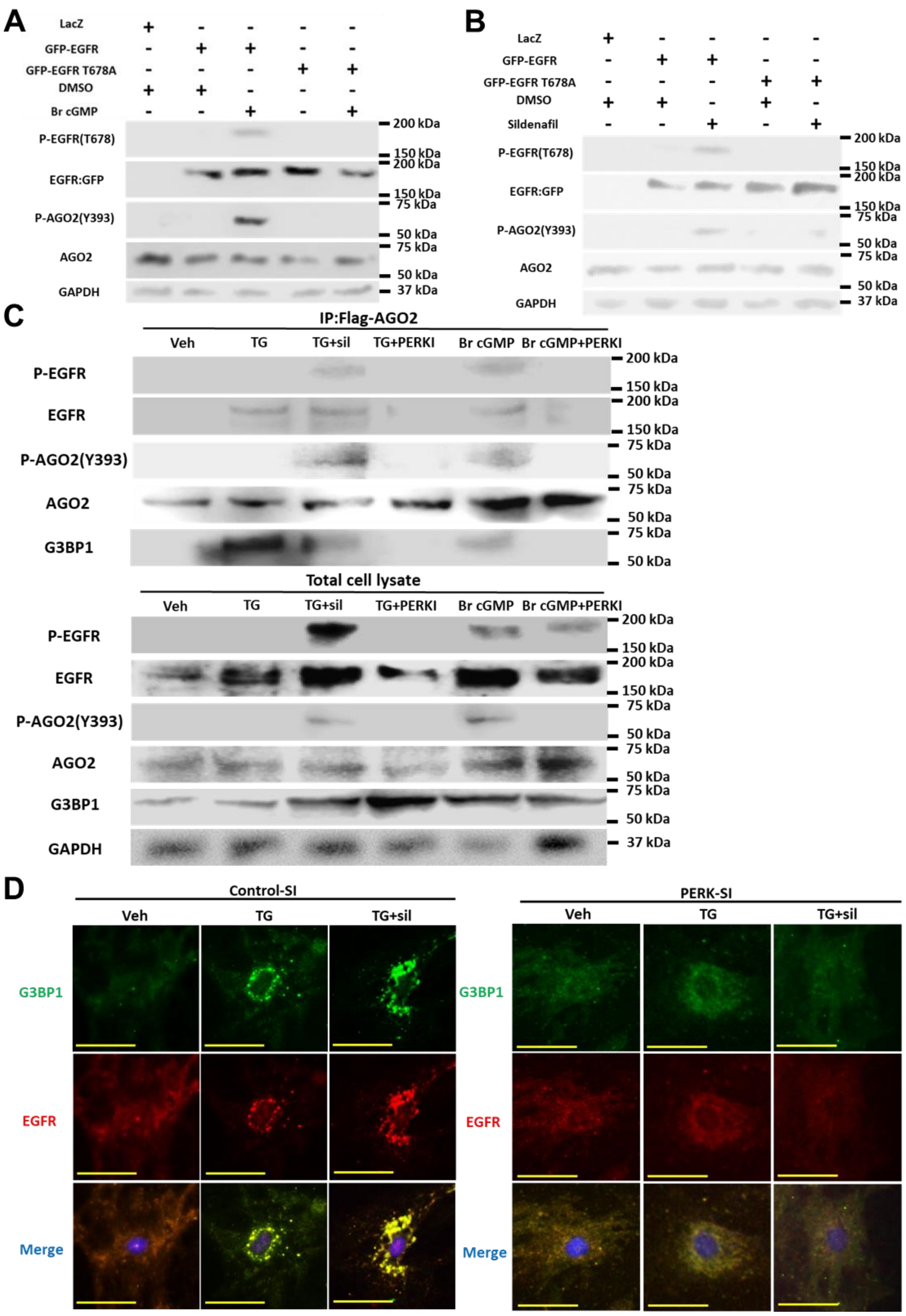


**Figure S2. PERK deletion suppressed p-AGO2 (Y393) by p-EGFR and AGO2–EGFR interaction.**

(AB) Western blot analysis of NRCMs transfected with plasmids of LacZ, GFP-tagged EGFR, and EGFR T678A, treated with or without Br cGMP (100µM, 30min, A) or sil (1µM 24hr, B).

(C) Immunoprecipitation and western blot analysis of NRCMs transfected with flag-tagged AGO2 plasmids.

(D) Control-SI or PERK-SI NRCMs, treated with vehicle (Veh), thapsigargin (TG, 1μM 24hr) with or without sildenafil (sil, 1μM 24hr). These cells were fixed and stained against EGFR (red), G3BP1 (green), and Hoechst (blue). Stress granules are indicated by the dense foci of G3BP1. Scale bar, 50 μm.

Figure S2. Related to Figure 1&5.
